# Supplementation with Probiotic Camel Milk Powder Improves Serum Glucose and Cholesterol as Well as the Related Cytokines in Patients with Type 2 Diabetes Mellitus

**DOI:** 10.3390/foods14193318

**Published:** 2025-09-24

**Authors:** Yue Liu, Ming Zhang, Ran Wang, Shaoyang Ge, Bing Fang

**Affiliations:** 1 School of Food and Health, Beijing Technology and Business University, Beijing 100048, China; 2 Department of Nutrition and Health, China Agricultural University, Beijing 100193, China

**Keywords:** *Bifidobacterium animalis*, camel milk, inflammation, adipokines, myokines, gut microbiota, fecal metabolites

## Abstract

Due to the close association between gut microbiota and diabetes, probiotic dairy products have drawn a lot of attention in the development of functional foods with anti-diabetic activity. In this study, 28 type 2 diabetic patients received 10 g of camel milk powder supplemented with *Bifidobacterium animalis* A6 (BBA6) twice a day, taking camel milk powder as the placebo. After 4 weeks of intervention, there was a significant decrease in fasting blood glucose, serum content of total cholesterol, and pro-inflammatory cytokines (IL-6, MCP-1). And, in the CA group, the level of irisin and osteocrin increased significantly, while the level of osteonectin also increased, but with no significance. For the adipokines, the intervention of CA decreased the adiponectin, resistin, lipocalin-2, and adipsin levels significantly. Gut microbiota analysis suggested a significant enrichment in the relative abundance of *Bifidobacterium* when compared with patients supplemented with camel milk powder alone. Furthermore, elevated fecal concentrations of glucose-1-phosphate, conduritol b epoxide, D-Arabitol, dehydroascorbic acid, and dl-p-Hydroxyphenyllactic acid, accompanied with a decrease in glycine, N-Acetylisatin, hydroxylamine, caprylic acid, maltotriose, and guaiacol, were found in patients of group CA. Compared with camel milk alone, the adding of BBA6 can significantly decrease fasting blood glucose in type 2 diabetic patients, while also improving dyslipidemia, chronic inflammation, and skeletal muscle functions, indicating the possibility of probiotic camel milk powder as a dietary treatment that targets metabolic syndromes such as diabetes.

## 1. Introduction

Diabetes is a serious, long-term condition that occurs when the body cannot effectively produce or use insulin. Type 2 diabetes mellitus (T2MD) accounts for around 90% of diabetes worldwide, which can be effectively prevented and managed through the adoption of healthy lifestyles, especially a healthy diet [1]. As a result, there are many studies focused on the evaluation of the hypoglycemic activity of functional foods or ingredients, which can be mainly divided into polyphenol-enriched plant-based foods [2,3,4,5] and proteins or peptides [6,7,8,9]. Meanwhile, since evidence has emerged about the close relationship between gut microbiota and diabetes [10,11], probiotics are used as a new effective therapeutic strategy in preventing and managing diabetes [12,13]. For example, Zhang et al. have proven that supplements of *Akkermansia muciniphila* can reduce the body weight and glycated hemoglobin of patients with T2DM [14]. Besides supplements with the strains themselves [15,16,17], probiotic dairy products such as probiotic soy milk [18] and probiotic fermented milk [19,20,21] were also found to improve the glycemic control of T2DM patients.

Camel milk is a rich source of vitamin C, lactic acid bacteria (LAB), beta-caseins, and milk whey proteins, including lactoferrin, lysozyme, lactoperoxidase, alpha-lactalbumin, and immunoglobulin [22]. And camel milk has a significant degree of biological activity. Compared with other milk, several studies reported the profound anti-diabetic activity of camel milk both in patients with type 1 diabetes [23,24,25,26,27] and T2MD [28,29,30]. However, camels mainly live in the desert areas of Africa/the Middle East or the cooler dry areas of Asia [31], leading to the unavailability of fresh camel milk for people living in other areas. Moreover, all the exiting clinical trials were based on fresh camel milk. It is not clear whether camel milk powder has a similar result.

*Bifidobacterium animalis* A6 (BBA6) was isolated from the feces of a centenarian. In our previous studies, it has been proven that BBA6 not only can promote mitochondrial biogenesis [32] but also enhanced fatty acid β-oxidation of adipose tissue [33] to alleviate obesity. Therefore, we speculated that BBA6 may have the potential to improve T2DM. Meanwhile, dairy products are important vectors for the delivery of probiotics to humans. And in order to solve the transportation and storage issues of camel milk, our lab developed a probiotic camel milk powder product. Therefore, the objective of this study was to evaluate the improving effect of camel milk power with BBA6 on T2DM patients. Except for some phenotypic indicators such as the glycemic index and serum insulin, some inflammatory factors, muscle factors, and adipokines were also detected. In addition, the changes in intestinal flora and bacterial metabolites of participants were also detected to analyze the possible mechanisms of BBA6 on improving T2DM.

## 2. Materials and Methods

### 2.1. Randomization, Blinding, and Intervention Protocol

This was a randomized, parallel, double-blind trial of T2DM patients, conducted in Beijing Chinese Medicine Hospital Pinggu Hospital, which lasted for 4 weeks. This study met the CONSORT criteria as recommended elsewhere [34]. The study was approved by the local ethics committee of China Agricultural University (CAUHR-2018026) and registered at ClinicalTrials.gov (NCT04296825).

The sample size determination and power analysis used by G* Power 3.1 program was based on a previous study [35]. The estimate sample size of 20 was calculated using the parallel clinical trial formula, assuming an alpha error of 0.05 and a power of 80%. Supposing an estimated 10% dropout rate, there were 22~23 patients for each group (45 patients in total).

A total of 45 T2DM patients were recruited from subjects attending the clinic of Beijing Chinese Medicine Hospital Pinggu Hospital. Based on previous research, the inclusion and exclusion criteria have been slightly modified and determined [14]. The inclusion criteria were as follows: (a) age of 35–68 years; (b) willingness to abstain from intake of all kinds of other milk, probiotic food, and fermented dairy products during the study, but otherwise sticking to previous eating habits. The exclusion criteria were as follows: (a) pregnancy or lactation in women; (b) patients with cancer; (c) allergy or intolerance to camel milk or cow milk. These criteria were verified during an inclusion visit that included a physical medical examination, dietary and physical activity assessments, standard anthropometrics, and an evaluation of fasting glycaemia, insulin, and lipid profile. After the verification, 40 subjects were eligible to participate in the study. Study procedure was explained to participants, and all participants provided written informed consent.

The 40 participants were randomly divided into two groups (20 individuals in each group): the camel milk+BBA6 group (CA group, camel milk powder with BBA6 at a dose of 2 × 10^10^ viable cells) and the control group (C group, camel milk). Both powders were packaged in the same bags (10 g each bag) and taken twice daily after breakfast and dinner, respectively, for 4 weeks.

All participants were asked to maintain their previous diet except for all kinds of other milk, probiotic food, and fermented dairy products, physical activity, and medications during the study. During the study, participants underwent interviews regarding adverse effects, symptoms, or changes in quality of life and diet every week. The allocation of groups was not revealed to the participants, or to the researchers who delivered probiotic camel milk or camel milk alone, or to those who conducted the weekly follow-ups.

The experimental design and sample collection time are shown in Table S1. The blood and feces samples were collected before consuming camel milk powder and BBA6 (W0) and after consuming for four weeks (W4). In addition, at W0 and W4, some indicators related to diabetes were tested, including fasting blood glucose, 2 h postprandial blood glucose, insulin, TG, TC, HDL-C, and LDL-C [36].

### 2.2. Camel Milk Powder and Probiotic Powder Preparation

The camel milk powder used in this study was provided by Xinjiang Jintuo Co., Ltd. (Urumqi, China) and packaged by Sanhe Fucheng Biological Technology Co., Ltd. (Langfang, China). The nutritional contents of camel milk powder used in this study are detailed in Supplementary Table S2.

The BBA6 was cultured in a liquid medium based on a previous study [32], which contained the following (per liter of deionized water): 10 g of beef extract, 5 g of yeast extract, 10 g of tryptone peptone, 20 g of glucose, 2 g of triamine citrate, 2 g of K_2_HPO_4_, 0.5 g of MgSO_4_, 5 g of sodium acetate, 0.5 g of L-cysteine, 0.25 g of MnSO_4_, and 1 mL of Tween-80. The chemicals and reagents used in the cultivation process were purchased from Beijing Aoboxing Biotechnology Co., Ltd. (Beijing, China). After counting and centrifugation, the bacterial cells were freeze-dried and mixed with the camel milk powder.

Patients were given sufficient supplies of the two products at the beginning of the intervention.

### 2.3. Blood Sample Collection and Measurements

Blood samples were collected twice at the beginning (W0) and the end (W4) of the study, respectively, and adhered to the standardized principle of fasting sample collection during collection [37]. On the day of blood sample collection, patients came to the hospital without breakfast; after the collection of the fasting blood samples, they were given the same breakfast, and the 2 h postprandial blood samples were collected after 2 h of the first bite of breakfast.

Human peripheral blood was collected in Vacutainer tubes (BD Biosciences, San Jose, CA, USA) and Vacutainer heparin tubes (BD Biosciences, San Jose, CA, USA), respectively. Blood samples were centrifuged at 1500× *g* for 30 min at room temperature. And within 1 h after blood collection, the fasting glycaemia, 2 h postprandial glycaemia, insulin, uric acid, and lipid were measured. The rest serum samples in Vacutainer heparin tubes were carefully removed, aliquoted, snap-frozen in liquid nitrogen, and stored in aliquots at −80 °C until further analysis.

Serum insulin was measured as in the previous study [38] by using the Architect i2000SR analyzer (Abbott Diagnostics, Abbott Park, IL, USA); blood glucose, content of total cholesterol (TC), total triglyceride (TG), high-density lipoprotein cholesterol (HDL-C), and low-density lipoprotein cholesterol (LDL-C) were measured using a Roche cobas ^®^ e 411 analyzers (Roche, Hvidovre, Denmark) according to the manufactures’ protocol by the certified core clinical laboratory at the Beijing Chinese Medicine Hospital Pinggu Hospital.

### 2.4. Cytokines and Hormones Assays

The Luminex multi factor detection technology was used to detect inflammatory factors, myokines, and adipokines [39]. For determination of inflammation cytokines [tumor necrosis factor-α (TNF-α), interleukin-6 (IL-6), monocyte chemotactic protein-1 (MCP-1)], myokines [fibroblast growth factor-21 (FGF-21), irisin, osteocrin, osteonectin], and adipokines (adiponectin, resistin, lipocalin-2, adipsin), the human cytokine immunobead panels (Milliplex, Millipore Saint Charles, MO, USA) coupled with a multiplex assay (Luminex, Thermo Fisher Scientific, Waltham, MA, USA)) were used according to the manufactures’ protocol.

### 2.5. Fecal Sample Collection and Gut Microbiota Analysis

Fecal samples were collected in the morning of the day of blood collection by patients themselves at home based on the previous study [40]. Before defecating, waterproof paper was first put into closestool to keep feces away from liquids, then a portion of feces was put into sterile tubes containing RNAlater (Qiagen, Hilden, Germany), and the other portion was put into empty sterile tubes. Some fecal samples were collected the day before blood collection due to a higher defecation frequency. Fecal samples were brought to the hospital in ice boxes and then stored at −80 °C.

DNA was extracted from fecal samples using the phenol–chloroform extraction method [41], quantified using a NanoDrop spectrophotometer (OneC, Thermo Fisher Scientific, Waltham, MA, USA), and stored at −80 °C until further analysis. DNA was amplified using the universal primers 338F (5′-ACTCCTACGGGAGGCAGCAG-3′) and 806R (5′-GGACTACHVGGGTWTCT AAT-3′) to target the V3–V4 region of bacterial 16S rRNA. The resulting 468 bp-sized products were assessed, quantified, pooled, and sequenced on an Illumina Miseq PE300 platform (Illumina, San Diego, CA, USA) at Shanghai Majorbio Bio-pharm Technology Co., Ltd. (Shanghai, China) using a paired-end sequencing strategy. Raw data were spliced, filtered, and then used to select the operational taxonomic units (OTUs) with USEARCH software (version 7.0) and a default cutoff of 97% sequence similarity.

OTUs were further subjected to the Ribosomal Database Project classifier software (version 2.14) for taxonomic identification, with an 80% confidence threshold at the phylum, class, order, family, genus, and species levels. Further analysis such as ANOSIM/Adonis tests, principal coordinates analysis, abundance heatmap, and differences in gut microbiome composition were analyzed on the free online platform of Majorbio I-Sanger Cloud Platform (https://cloud.majorbio.com/) using weighted unifrac distance matrices.

### 2.6. Fecal Metabolomics Analysis

The untargeted metabolomics analysis of feces was conducted based on previous research, and some modifications have been made [42]. The 50 milligrams of feces were mixed with 40 μL internal standard (0.3 mg/mL, 2-chloro-L-phenylalanine), dissolved in methanol, and ultrasonically extracted with 360 μL methanol, then with 200 μL chloroform and 400 μL ddH_2_O in an ice bath for 30 min, respectively. After extraction, samples were centrifuged at 12,000 rpm at 4 °C for 10 min. Then, 400 μL supernatant was volatilized and oximated with 80 μL methoxyamine hydrochloride in pyridine (15 mg/mL), and they were shaken for 90 min at 37 °C, after which they were trimethylsilylated by adding 30 μL BSTFA (containing 1% TMCS) and 20 μL n-hexane, incubating for 1 h at 70 °C. After being left at room temperature for 30 min, samples were then subjected to GC-MS analysis. The chemicals used in this part were chromatographic grade and purchased from Thermo Fisher (Waltham, MA, USA).

Metabolic profiling of fecal samples was acquired by an Agilent 7890 A/5975C GC-MS (Agilent Technologies, Santa Clara, CA, USA) using a HP-5MS fused silica capillary column (30 m × 0.25 mm × 0.25 μm, Agilent J&W Scientific, Folsom, CA, USA). A total of 1 μL of the sample was injected in a non-split mode. The injector, ion source, and quadrupole rod temperatures were 260 °C, 230 °C, and 150 °C, respectively. High-purity helium (>99.999%) was used as the carrier gas, with a flow rate of 1.0 mL/min. The GC oven temperature program consisted of 60 °C for 2 min, after which the temperature ramped to 310 °C at 8 °C/min and held steady for 6 min. Mass spectra were acquired, and the mass scan range was set at *m*/*z* 50–600. Fecal samples were analyzed randomly.

Raw GC-MS mass spectra were converted to CDF format files by ChemStation (version E.02.02.1431, Agilent, CA, USA) and subsequently preprocessed using Chroma TOF (version 4.34, LECO, St Joseph, MI, USA), including raw signal extraction, data baseline filtering, peak identification, and integration. After alignment with the statistical comparison component, the “.CSV” file was obtained with three-dimension data sets including sample information, retention time, the mass-to-charge ratio, and peak intensity. Identification of metabolites was conducted using the Automatic Mass Spectral Deconvolution and Identification System, which was searched against commercially available databases such as the National Institute of Standards and Technology and Fiehn libraries. The signal integration area of each metabolite was normalized to the internal standard (2-chloro-L-phenylalanine) for each sample.

The normalized data were transformed using SIMCA-P 14.0 software (Umetrics AB, Umea, Sweden) for principal component analysis and partial least Squares-discriminant analysis (PLS-DA). The variable importance in projection (VIP) values of all the metabolites from the PLS-DA model were taken as criteria to find the variable importance of differential metabolites, and variables with a VIP >1.0 and a *p*-value  <  0.05 were considered relevant for group discrimination. The statistical significance between two groups was evaluated by a univariate Student’s t-test.

### 2.7. Statistical Analysis

Data entry was performed twice by two separate people. Differences between W0 and W4 of each group were evaluated by paired two-tailed Student’s *t*-tests using GraphPad Prism version 7.0 software (San Diego, CA, USA). Differences between the two groups at the same time point (W0 or W4) were compared by unpaired two-tailed Student’s *t*-tests using GraphPad Prism. Statistical significance was evaluated at an alpha level of 0.05.

## 3. Results and Discussion

### 3.1. Study Population

**Figure 1:**
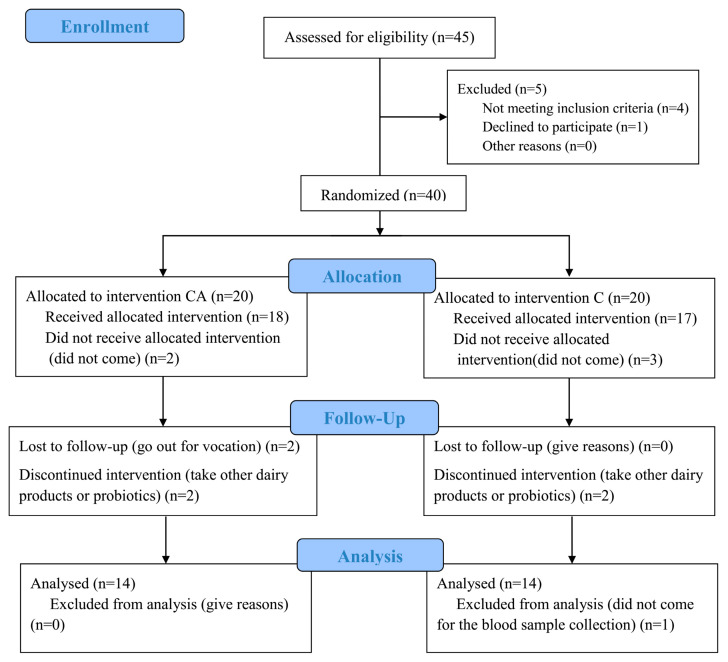
Flow diagram.

As shown in Figure 1, of the 40 participants that were randomized, 5 people did not come to pick up the intervention products, 2 people were lost to follow-up due to going out for travel, and 5 people were poor compliances (took other dairy products and probiotics). At the end of the 4-week intervention, 28 participants completed the experiment and were subjected to the analysis: 14 received camel milk powder supplemented with BBA6 (CA) and 14 received camel milk powder (C). None of the participants reported any adverse effects, including gastrointestinal disorders. Baseline comparison showed no significant differences in blood glucose, insulin, and lipid profiles between different groups (*p* > 0.05, Table 1).

### 3.2. Changes in Glycemic Indices and Serum Insulin

**Figure 2:**
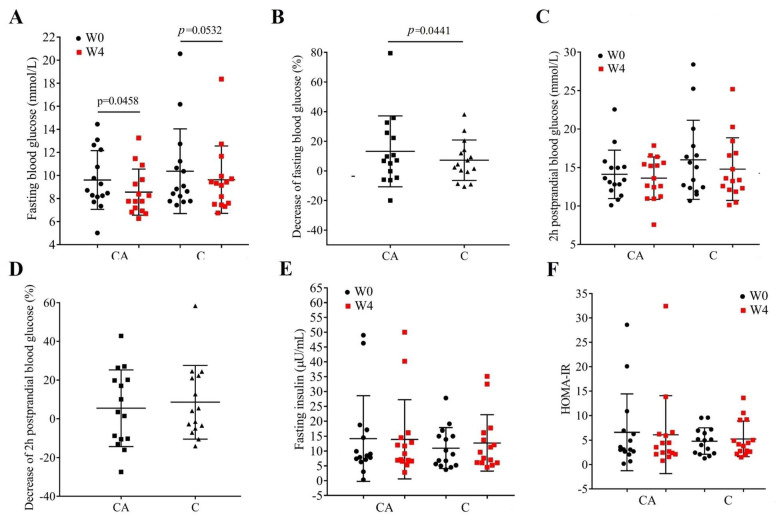
Fasting blood glucose, 2h postprandial blood glucose, fasting serum insulin, and HOMA-IR in each group before (W0) and after (W4) the intervention. (**A**), fasting blood glucose; (**B**), decrease in fasting blood glucose (squares: CA group; triangles: C group); (**C**), 2h postprandial blood glucose; (**D**), decrease of 2h postprandial blood glucose (squares: CA group; triangles: C group); (**E**), fasting serum insulin; and (**F**), HOMA-IR in patients treated with camel milk (C group) and camel milk supplemented with BBA6 (CA group).

The fasting blood glucose, 2 h postprandial blood glucose, and fasting serum insulin of patients before and after the 4-week intervention are shown in Figure 2. At baseline, there were no significant differences between the two groups (*p* > 0.05). The hypoglycemic effect of camel milk has been proven in type 1 [24,25,26,27] and type 2 diabetic patients [28,29,30]; in this study, patients in the CA group exhibited a significant decrease in fasting blood glucose after the intervention compared with C group (*p* = 0.0458, Figure 2A) and a more effective hypoglycemic activity than camel milk powder alone (*p* = 0.0441, Figure 2B). Similarly, Alharbi et al. also found that fermented camel milk can effectively reduce the elevated blood sugar levels in Streptozotocin-induced diabetes in rats [43], which further proves our results that the combination of probiotics and camel milk exhibits better hypoglycemic effects. However, there were no significant changes in the 2 h postprandial blood glucose of patients either before and after the intervention (Figure 2C) or between the two groups (Figure 2D). This is different than Agrawal’s study: they found that, after the intervention of camel milk, the patients with T2DM from Iran showed a decrease in postprandial glucose [44]. We guess that the reason for this difference may be due to different dietary habits in the two regions.

The serum content of insulin was also not affected (Figure 2E, *p* > 0.05), and the intervention did not improve the insulin resistance of the patients (Figure 2F, *p* > 0.05). Previous studies on camel milk found a consistent unchanged insulin level in type 1 diabetic patients [24,45,46,47,48], but in T2DM patients the existing results were inconsistent [28,29], which may be due to the complicated mechanism in T2DM.

### 3.3. Changes in Lipid Profile and Cardiovascular Risk

**Figure 3:**
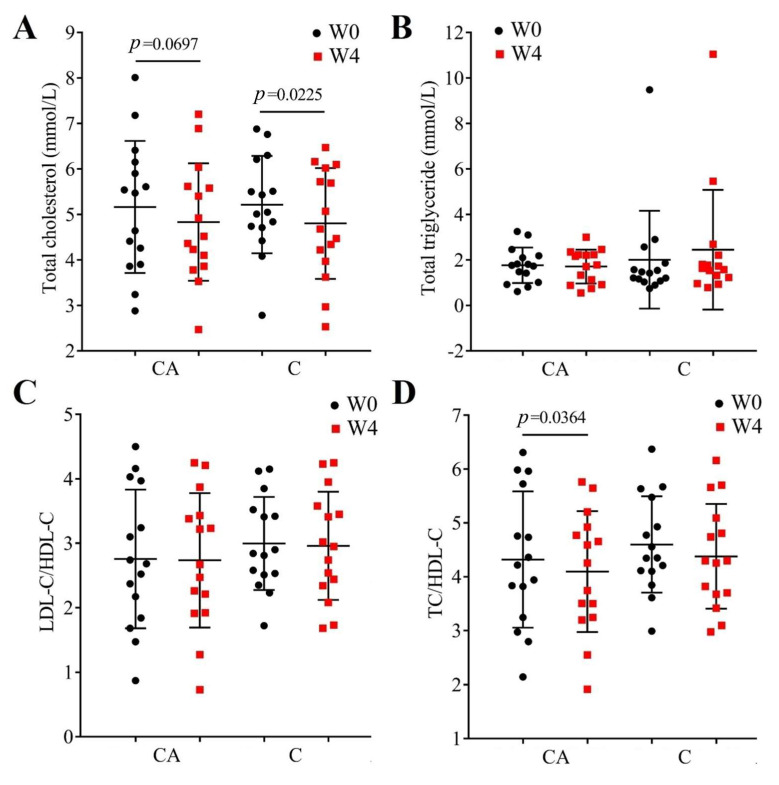
Lipid profile in each group before (W0) and after (W4) the intervention. (**A**), total cholesterol; (**B**), total triglyceride; (**C**), the LDL cholesterol/HDL cholesterol ratio; (**D**), total cholesterol/HDL-cholesterol ratio in patients treated with camel milk (C) and camel milk supplemented with BBA6 (CA).

The relationship between diabetes and atherosclerotic cardiovascular disease is well established, with a significantly elevated risk for cardiovascular disease in diabetic patients [49]; therefore, we also measured the serum content of TC, TG, and the indicators of vascular risk (LDL/HDL cholesterol ratio and TC/HDL-C, Figure 3). As we can see from the results, both at baseline (W0) and post-intervention (W4), there were no significant differences between the two groups (CA-W0 vs. C-W0 and CA-W4 vs. C-W4, *p* > 0.05), whereas, after the intervention of CA, the TC showed a decreasing trend but was not significant (*p* = 0.0697, Figure 3A), but in the C group the TC significantly decreased (*p* = 0.0225). Furthermore, although there was no change in TG (Figure 3B) and the decreased TC in group CA was nonsignificant (*p* = 0.0697, Figure 3A), the intervention of CA resulted in a significant decrease in the ratio of TC and HDL-C (TC/HDL-C, *p* = 0.0364, Figure 3D), indicating a decreased vascular risk. However, there was no significant change in TC/HDL-C in the C group (Figure 3D). Previous clinical studies seldomly reported the effects of camel milk on the lipid profile, although animal studies reported a consistent decrease in TC [23,50,51]. In the limited studies in T2DM patients, Hanieh et al. found that patients who consumed 200 mL of camel milk for two months did not show a significant difference in blood lipids [52]. But, Sboui et al. proved that a supplement with 500 mL of camel milk can decrease the TC and TG significantly [30]. And, for the probiotic camel milk, some animal experiments have reported a decrease in TC and TG [53,54], which differs with our results. In these articles, the authors mixed the camel milk and the strains for fermentation. In this way, the probiotics may use the special nutrients in camel milk to produce unique metabolites, resulting in better probiotic effects. It is interesting that the effect of *B. animal* A6 on improving obesity has been reported in previous studies; we speculate whether mixed fermentation is necessary for better results.

### 3.4. Changes in Inflammatory Cytokines

**Figure 4:**
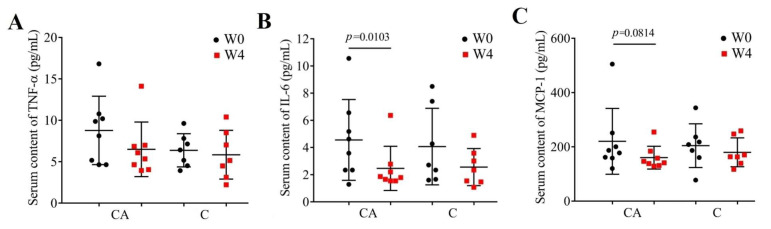
Serum contents of inflammatory cytokines in each group before (W0) and after (W4) the intervention. (**A**), TNF-α; (**B**), IL-6; (**C**), MCP-1 in patients treated with camel milk (C) and camel milk supplemented with BBA6 (CA).

It was reported that there was a chronic inflammation in diabetes [55] and a greater antioxidant and immunomodulatory activity of camel milk protein than bovine and other whey proteins [56,57]. So, in order to test whether probiotic camel milk has better anti-inflammatory effects, the content of TNF-α, IL-6, and MCP-1 were detected. As shown in Figure 4, there were no significant differences in the serum contents of inflammatory markers (TNF-α, IL-6, MCP-1) between groups both at baseline and post-intervention (*p* > 0.05). Within-group comparisons (W0 vs. W4) suggested that the decreased TNF-α (*p* > 0.05), IL-6 (*p* = 0.0103) and MCP-1 (*p* = 0.0814) contents in all CA groups were more obvious than those in C groups. Previous studies found that camel milk or camel milk whey proteins can reduce the pro-inflammatory IL-1β, IL-6, and TNFα in diabetic rats [29,58,59]. However, there are few or no studies proving that camel milk has a clinical effect on reducing inflammatory factors.

### 3.5. Changes in Adipokines and Myokines Profile

**Figure 5:**
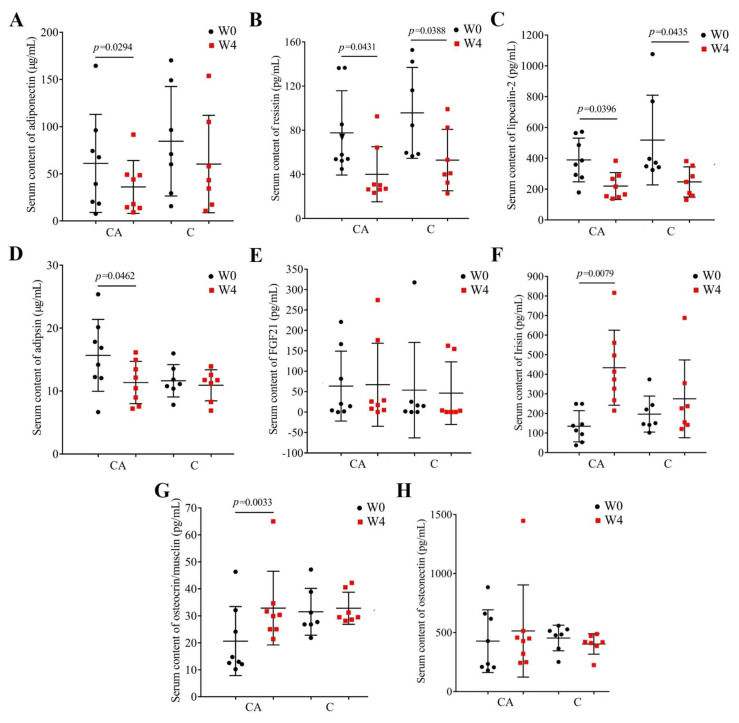
Serum contents of adipokines and myokines in each group before (W0) and after (W4) the intervention. (**A**), adiponectin; (**B**), resistin; (**C**), lipocalin-2; (**D**), adipsin; (**E**), FGF-21; (**F**), irisin; (**G**), osteocrin; (**H**), osteonectin in patients treated with camel milk (C) and camel milk supplemented with BBA6 (CA).

T2DM is the most common metabolic disease at present, and insulin resistance is considered to be the main reason for the occurrence and development of T2DM [60]. Lots of research has shown that the impaired function of muscles and adipose tissue is an important cause of insulin resistance. Skeletal muscle and adipocytes are secretory organs, which communicate with each other to regulate energy homeostasis and insulin sensitivity though the cytokines, called myokines and adipokines, respectively [61,62]. Therefore, we measured the serum contents of adipokines (adiponectin, resistin, lipocalin-2, adipsin) and myokines (FGF-21, irisin, osteocrin, osteonectin) in patients before and after the 4-week intervention to understand whether these interventions improved the function of patients’ skeletal muscle and adipose tissue, and the results are shown in Figure 5. There were no significant differences between different groups at baseline (W0) and after intervention (W4, *p* > 0.05). In the C group, results showed that it can significantly decrease the levels of resistin and lipocalin-2 (Figure 5B,C). Intervention with camel milk powder supplemented with BBA6 significantly decreased the content of adipokines (adiponectin, resistin, lipocalin-2, and adipsin) and increased myokine (irisin and osteocrin) levels. Although increased levels of adiponectin (Figure 5A) and adipsin (Figure 5D) were found to be associated with a lower risk of T2DM [63,64] in humans and improvement in pancreatic beta-cell function in mice [64,65], respectively, we found a significant decrease in patients with a significant decrease in fasting blood glucose (group CA). This further explains the relationship between adipose tissue dysfunction and diabetes. The other two adipokines, resistin (Figure 5B) and lipocalin-2 (Figure 5C), which decreased significantly in patients treated with camel milk powder alone and in combination, were reported to be good for the improvement of diabetes [66]. Similarly, Auguet et al. found that, in severely obese women, lipid carrier transporter 2 is upregulated and accompanied by an increase in pro-inflammatory cytokines [67]. Elevated serum lipocalin-2 is closely and independently associated with impaired glucose regulation and T2DM in Chinese people [68], and a lipocalin-2 deficiency attenuates the insulin resistance associated with obesity in mice [69]. Resistin promotes insulin resistance in mice, whereas whether it does so in humans is unclear [70,71], since it was synthesized in adipocytes in mice, whereas in humans it is generated by macrophages and monocytes and not adipocytes [72].

As for the myokines, no significant changes were observed in FGF21 in the two groups. And after intervention of CA, the osteonectin showed an increasing tendency, while showing a decreasing tendency in C group, although not significant (Figure 5H). Furthermore, the significant and specific decreases in irisin (*p* = 0.0079, Figure 5F) and osteocrin (*p* = 0.0033, Figure 5G) were also an indicator of the improvement of diabetes. Irisin is an adipo-myokine hormone produced during exercise. Studies have proven that irisin exhibits anti-inflammatory effects by inhibiting NF-κB signaling and countering insulin resistance [73]. Meanwhile, Irisin elevates in healthy states but reduces in diseases; this is the same as our results [74]. Osteocrin is also a regulator of bone growth as a novel vitamin D-regulated bone-specific protein [75]. Zhang et al. found that osteocrin can prevent diabetic cardiomyopathy via restoring proteasomal activity [76]. In this study, the significant increase in these two myokines in patients treated with camel milk powder supplemented with BBA6 indicated an improvement in diabetes.

### 3.6. Changes in Gut Microbiota

**Figure 6:**
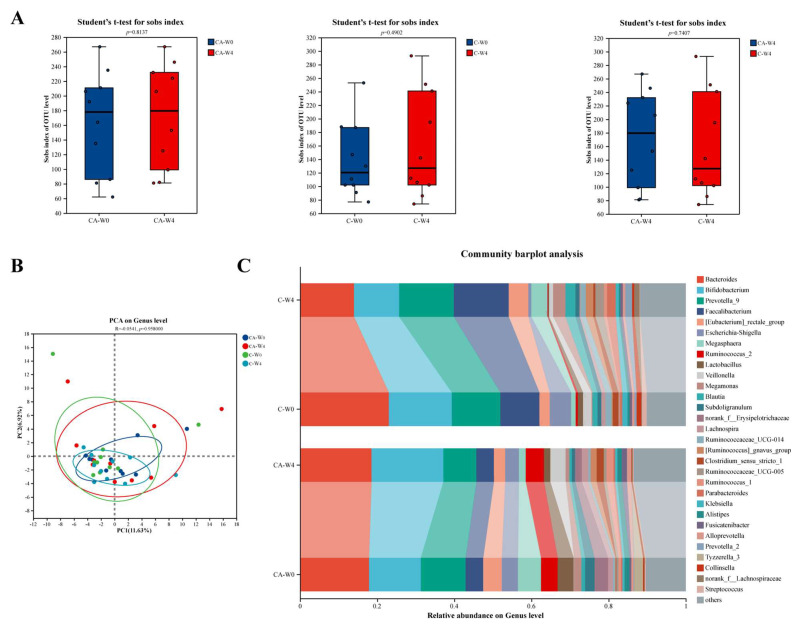
Changes in microbial composition after intervention of camel milk (C) and camel milk supplemented with BBA6 (CA). (**A**) α diversity; (**B**) PCA analysis; (**C**) Species composition at the genus level.

More and more evidence suggests a close relationship between gut microbiota and diabetes [77], and, since there is a component of probiotics in our study, we analyzed gut microbiota before and after the intervention (Figure 6). In Figure 6A, although there was no significant change in diversity after C and CA interventions, the species richness in the CA group was higher than that in the C group based on the post-intervention results. As shown in Figure 6B, the PCoA analysis showed that, after the interventions of C and CA, there was no significant difference in the species composition between the two groups. Such inconspicuous results have also been found by Qin et al. in the gut microbiota of healthy individuals and patients with T2DM [78]. Therefore, they believed that the gut microbiota of patients with T2DM is imbalanced, but it is a moderate imbalance, not a severe one. Thus, the reason for these results may be due to the individual differences and the short intervention time. To further observe the changes in gut microbiota before and after intervention, the community composition at the genus level is shown in Figure 6C. From the result, it was found that the interventions of C and CA have different regulatory trends on various microorganisms. The *Bacteroides* and *Bifidobacterium* are proven to have a lower abundance in the intestinal flora of patients with T2DM [79]. And their abundance increased after the intervention of CA, but decreased in group C. This may suggest that CA intervention has a potential role in improving diabetes. And for the characteristic bacteria of T2DM, the abundance of *Lactobacillus* decreased in both groups.

**Figure 7:**
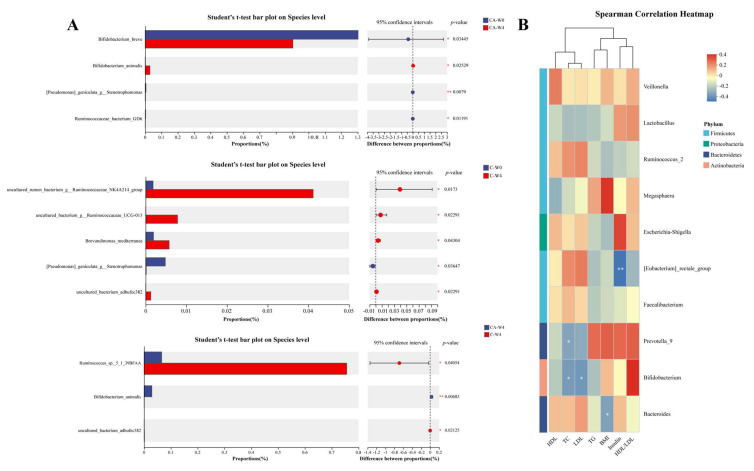
Different gut bacteria at the genus level in patients intervened with camel milk (C) and camel milk supplemented with BBA6 (CA) by two-tailed Student’s *t*-test (**A**) and correlation analysis between flora and physiological indexes of diabetes (**B**).

To further observe the changes in gut microbiota, we analyzed the microorganisms with significant differences before and after intervention by using Student’s t-test. As shown in Figure 7A, after the intervention of CA, the abundance of *Bifidobacterium breve* decreased as the *Bifidobacterium animals* increased. This is contrary to some research findings. For example, Chaiyavat et al. found that supplementing with *B. brevis* can prevent the deterioration of representative clinical parameters in T2DM subjects and change the components of the gut microbe [80]. On the contrary, a mate analysis study showed that the *Lactobacillus* which enrich in T2DM were positive with the *B. brevis* [81]. Therefore, we speculated that these results may be caused by the diversity and complexity of human gut microbiota. The increase of *B. animalis* effectively explains the colonization of BBA6. Meanwhile, we also observed that the C group increased the abundance of *Ruminococcus*, which play an important role in digesting resistant starch. Studies have proven that more consumption of resistant starch can improve T2MD, and the gut microbiota can guide the effects of it in improving T2MD [82,83]. And the *Ruminococcus* are the productor of butyrate, which can protect the pancreatic islet cells and regulate blood sugar [84]. Thus, we speculated that C and CA can change the digestive mode of subjects.

Then, we investigated whether gut microbes were associated with biochemical indicators of T2MD, and the results are shown in Figure 7B. Results showed that the *Bacteroides* were significantly negatively correlated with BMI. And the *Bifidobacterium* and *Prevotella-9* were evidently negatively correlated with TC, while the *Bifidobacterium* also negatively correlated with LDL. For the content of insulin, the *[Eubacterium]_rectale group* showed an obviously negative association with it. Based on the difference test, we found that *Ruminococcus* are related to the effects of C and CA in improving T2MD. Figure 7B shows that *Ruminococcus_2* is positively correlated with TC and LDL, and has a high correlation coefficient. As for the *Bifidobacterium*, it has the highest correlation coefficient with the HDL/LDL, which may indicate its potential mechanism for improving T2MD.

### 3.7. Changes in Fecal Metabolites

**Figure 8:**
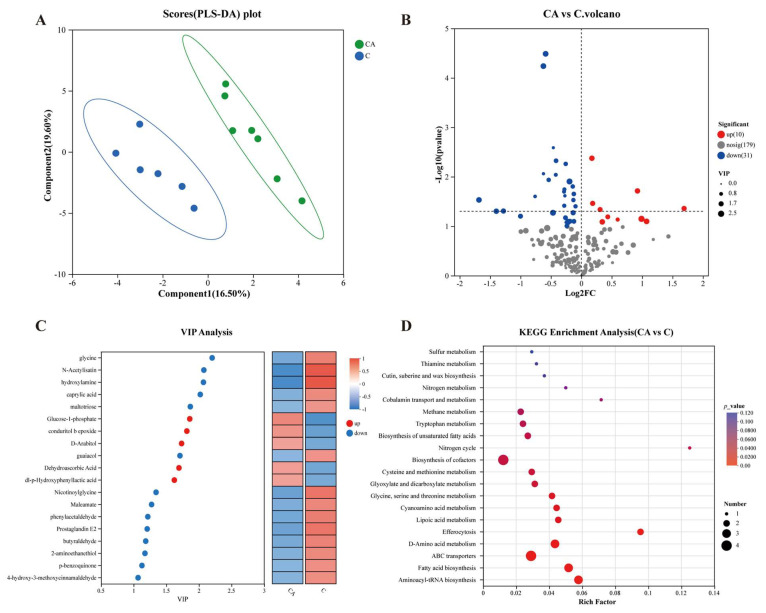
Analysis of differential metabolites in feces in camel milk (C) and camel milk supplemented with BBA6 (CA). (**A**) PLS-DA analysis, (**B**) differential metabolite analysis, (**C**) VIP analysis, (**D**) KRGG enrichment pathway analysis of differential metabolites.

Figure 8 summarizes the change in metabolomics in fecal samples from participants with subjects after the intervention of CA and C. From the results, we can see that the PLS-DA analysis (Figure 8A) showed an obvious separation between the two groups, while the separation rate of two components are 19.60% and 16.50%, respectively. These indicated that the CA group drove a distinct metabolic signature. Then, the volcano analysis was used to detect the differential metabolites. A total of 220 metabolites were identified, and among them 10 metabolites were significantly upregulated and 31 downregulated in the CA group (Figure 8B). After that, 19 metabolites were regarded as key metabolites and are shown in Figure 8C. Among these key metabolites, those with a VIP value greater than 1.5 were analyzed especially. N-Acetylisatin and glycine are precursor substances of glutathione, which is regarded as an antioxidant synthesized by glycine and can protect the function of β cells [83,85].Tuell et al. found that dietary supplementation with glycine can achieve lower level of oxidative stress and oxidant damage in patients with T2MD through an increase in the synthesis of glutathione [86]. And Lei et al. proved that the N-Acetylisatin inhibits NADPH oxidase activation in diabetes and attenuates tissue oxidative damage in all organs. In this study, we observed a decrease in N-Acetylisatin and glycine, which may indicate the enhancement of antioxidant ability. For the hydroxylamine, Kimura et al. found that it can enhance the glucose uptake of C2C12 cells through the PIK3 pathway [87]. We also found a decrease in it in the CA group. Caprylic acid is a medium chain fatty acid (MCFA), which has been proven to be associated with reduced inflammation and improved insulin sensitivity in vitro [88]. Maltotriose [89] and glucose-1-phosphate [90] have been proven to be higher in T2MD patients than in healthy controls. We found a decrease in maltotriose but an increase in glucose-1-phosphate in the CA group. Studies proved that conduritol B epoxide and D-Arabitol are potential treatments for T2DM. Conduritol B epoxide is an irreversible inhibitor of β-glucosidase and can block the hydrolysis of sugar [91]. And D-Arabitol can improve systemic insulin sensitivity by reshaping the gut microbiota [92]. In this article, we observed an increase in both metabolites in the CA group. Previous studies have shown that people with T2DM have lower plasma dehydroascorbic acid concentrations than those with normal glucose control [93,94]. This research also found that, after the intervention of CA, the concentration of dehydroascorbic acid was up. In order to further explore the pathways of CA’s improvement of T2DM, we conducted a KEGG pathway enrichment analysis on differential metabolites (Figure 8D). The results showed that the CA group preferentially activated sulfur metabolism (*p* = 0.020), glycine/serine/threonine metabolism (*p* = 0.040), and thiamine metabolism (*p* = 0.080). These pathways converge on the generation of glutathione [86] and one-carbon units [95]—both essential for countering oxidative stress and enhancing insulin signaling.

## 4. Conclusions

As a traditional milk in Africa, Asia, and the Middle East, it was believed that the regular consumption of fresh camel milk may aid in the prevention and control of diabetes. In previous studies, we investigated the improvement effect of camel milk in improving T2MD. We found that, compared with cow’s milk, the supplement with camel milk can decrease fasting blood glucose, 2 hr postprandial blood glucose, serum content of total cholesterol, resistin, and lipocalin-2. And content of osteocrin, amylin, and GLP-1 showed a significant increase in the camel milk group. The gut microbiota has also changed; the camel milk powder supplement significantly enriched the relative abundance of *Clostridium_sensu_stricto_1* and *[Eubacterium]_eligens_group* [28].

And, in this study, we evaluated the anti-diabetic activity of a probiotic camel milk product, camel milk powder supplemented with BBA6, a strain of Bifidobacterium animalis isolated by our lab from the feces of Longevity Elderly in Bama, Guangxi. Compared with the camel milk, the probiotic camel milk product showed a better improving effect on blood glucose, blood lipids, adipokines, myokines, and the gut microbiota after the 4-week intervention. These results in combination suggested that the probiotic camel milk product can be used as part of the treatment of T2MD.

However, as a clinical trial, this study inevitably has some limitations. First of all, due to the high dropout rate in this study, the sample size was relatively small; therefore, in subsequent experiments, we will expand the sample size to further validate the experimental results. In addition, the probiotics used in this experiment were from a directly freeze-dried powder added into camel milk powder instead of mixed during fermentation. Thus, it is necessary to further explore the improvement effect of mixed fermentation on T2DM patients.

## Figures and Tables

**Table 1:** Baseline characteristics of study participants.

Parameters	CA	C
Number (female/male)	14 (10/4)	14 (8/6)
Age (years)	58.36 ± 6.25	57.29 ± 7.57
BMI (kg/m^2^)	26.56 ± 4.40	26.56 ± 4.40
Fasting blood glucose (mmol/L)	9.39 ± 2.50	10.48 ± 3.78
2 h postprandial blood glucose (mmol/L)	14.05 ± 3.26	16.19 ± 5.28
Insulin (μU/mL)	11.69 ± 11.12	11.01 ± 7.11
TG (mmol/L)	5.20 ± 1.50	5.10 ± 1.00
TC (mmol/L)	1.77 ± 0.81	1.14 ± 0.14
HDL-C (mmol/L)	1.24 ± 0.26	1.22 ± 0.26
LDL-C (mmol/L)	3.27 ± 1.25	3.33 ± 0.86
TC/HDL-C	4.29 ± 1.31	4.47 ± 0.77

Notes: Data were analyzed for normal distribution by IBM SPSS Statistics 20.0 and expressed as mean ± S.D; C (camel milk), CA (camel milk with BBA6).

## Data Availability

The original contributions presented in the study are included in the article/Supplementary Material; further inquiries can be directed to the corresponding author.

## Supplementary Materials

The following supporting information can be downloaded at https://www.mdpi.com/article/10.3390/foods14193318/s1. Table S1: Summary of testing indicators and methods, Table S2: Nutritional contents of camel milk powder.

## Abbreviations

The following abbreviations are used in this manuscript:

BBA6	*Bifidobacterium animalis* A6
T2DM	Type 2 diabetes mellitus
W0	The beginning of the study
W4	The end of the study
CA	Camel milk supplemented with BBA6
C	Camel milk
TC	Total cholesterol
TG	Total triglyceride
HDL-C	High-density lipoprotein cholesterol
LDL-C	Low-density lipoprotein cholesterol
TNF-α	Tumor necrosis factor-α
IL-6	Interleukin-6
MCP-1	Monocyte chemotactic protein-1
FGF-21	Fibroblast growth factor-21
OTUs	Operational taxonomic units
PLS-DA	Squares-discriminant analysis
VIP	Variable importance in projection
