# Supplementary material for: Supplementation with Probiotic Camel Milk Powder Improves Serum Glucose and Cholesterol as Well as the Related Cytokines in Patients with Type 2 Diabetes Mellitus
Source: Foods. 2025 Sep 24;14(19):3318. doi: 10.3390/foods14193318 (PMC12524154; doi:10.3390/foods14193318)
Supplement: Supplementary file 1 [file foods-14-03318-s001.zip › foods-3860234-supplementary.pdf]

Table S1. Summary of Testing Indicators and Methods

Testing index	Test method	Testing party	Sample source
Serum insulin	Architect i2000SR analyzer	Beijing Chinese Medicine Hospital Pinggu Hospital	Blood sample
Blood glucose, TC, TG, HDL-C, LDL-C	Roche cobas ® e 411 analyzer	Beijing Chinese Medicine Hospital Pinggu Hospital	Blood sample
Inflammation cytokines (TNF-α, IL-6, MCP-1)	MILLIPLEX MAP Human Metabolic Hormone Magnetic Bead Panel (Luminex)	-	Blood sample
Myokines (FGF-21, irisin, osteocrin, osteonectin)	MILLIPLEX MAP Human Myokine Magnetic Bead Panel (Luminex)	-	Blood sample
Adipokines (adiponectin, resistin, lipocalin-2, adipsin)	MILLIPLEX MAP Human Adipokine Magnetic Bead Panel 1 (Luminex)	-	Blood sample
Gut microbiota	Bacterial 16S rRNA	Shanghai Majorbio Bio-pharm Technology Co. Ltd.	Fecal samples
Metabolomics analysis	GC-MS	Shanghai Majorbio Bio-pharm Technology Co. Ltd.	Fecal samples

Table S2. Nutritional contents of camel milk powder.

Parameters	Pre 100g
Energy	2359KJ
Fat	35.9g
Protein	25.2g
Carbohydrate	35.4g
Na	419mg
